# Association of cigarette production and tobacco retailer density on secondhand smoke exposure in urban China

**DOI:** 10.1136/tobaccocontrol-2021-056655

**Published:** 2021-07-06

**Authors:** Connie Hoe, Joanna E Cohen, Tingzhong Yang, Sihui Peng, Weifang Zhang

**Affiliations:** 1 International Health Department, Johns Hopkins University Bloomberg School of Public Health, Baltimore, Maryland, USA; 2 Institute for Global Tobacco Control, Johns Hopkins University Bloomberg School of Public Health, Baltimore, Maryland, USA; 3 Women's Hospital/Center for Tobacco Control Rsearch, Zhejiang University School of Medicine, Hangzhou, Zhejiang, China; 4 School of Medicine, Jinan University, Guangzhou, Guangdong, China; 5 Stomatology Hospital, Zhejiang University School of Medicine, Hangzhou, Zhejiang, China

**Keywords:** secondhand smoke, advertising and promotion, environment, low/middle income country, tobacco industry

## Abstract

The primary aims of this study are to examine the associations between two key environmental factors—regional cigarette tobacco production and tobacco retail outlet density—and secondhand smoke (SHS) exposure in urban China and to explore the possible mechanisms that explain this association.

**Methods:**

A cross-sectional multistage sampling design was used to collect individual information in 21 cities in China. Environmental variables were retrieved from national databases. Multilevel logistic regression analysis was conducted to examine the associations between regional cigarette tobacco production, tobacco retail outlet density and SHS exposure. Structural equation modelling was employed to determine possible mechanisms.

**Results:**

SHS exposure prevalence defined as daily exposure to SHS for at least 15 min/day at the time of the survey was found to be 28.1% among non-smokers (95% CI 27.1 to 29.0) across the 21 cities. The multilevel logistic regressions showed that province-level per capital cigarette production (OR: 2.72 (95% CI 1.56 to 4.76)and per GDP cigarette production(OR:1.69(95% CI 1,42,2.01), and city-level tobacco retail outlet density (OR: 2.66 (95% CI 1.63 to 4.38)) were significantly associated with SHS exposure. Moreover, results showed that these associations may be explained by the level of tobacco advertisement, which influences social norms, including attitudes and behaviours toward SHS exposure.

**Conclusions:**

Findings shed light on the role of cigarette manufacturers and retailers in producing environmental SHS pollution. To address the health and economic burden associated with SHS in China, it will be critical for the Chinese government to enact tobacco control measures consistent with the Framework Convention for Tobacco Control. Efforts should also focus on restricting the permitted density of tobacco retail outlets, and tobacco production in China.

## Background

Secondhand smoke (SHS), also known as tobacco smoke pollution or passive smoke, is produced from (1) the burning of cigarettes and other tobacco products and (2) smoke exhaled by a smoker.^1^ SHS contains chemicals that contribute to environmental pollution and many studies have clearly linked SHS exposure to adverse health consequences in non-smokers, including lung cancer, heart disease and asthma in children.^2–4^ Despite these grave consequences, a large percentage of the world is still exposed to SHS. It was estimated that globally about one-fifth of males and one-third of women were exposed to SHS and that this exposure caused almost 900 000 deaths in 2016.^5^

China is the largest producer and consumer of tobacco products in the world, with about 300 million smokers and 740 million SHS victims.^6^ The 2018 China Global Adult Tobacco Survey showed that the prevalence of SHS exposure among non-smokers is high (68.1%).^7^ Further, it is estimated that over 100 000 people are killed each year as a result of SHS.^6^ While the country ratified the Framework Convention for Tobacco Control (FCTC) in 2005, it still does not have a national smoke-free law as per Article 8 of the FCTC. A number of subnational jurisdictions in China have developed and implemented smoke-free policies in recent years. Beijing, Lanzhou, Nanjing, Qingdao, Shanghai, Shenzhen and Tangshan, for example, all have comprehensive smoke-free policies that ban smoking in indoor public and workplaces.^8^ Studies, thus far, have shown positive results. In Beijing, for example, smoking reduced in restaurants after the Beijing Smoking Control Regulation took effect in 2015; smoking was observed in 40.3% of restaurants before the law took effect and 14.8% of restaurants 1 month after the law took effect.^9^ Sansone *et al* also found strong public support in China for comprehensive smoke-free policies.^10^ Yet, the adoption of tobacco control laws has been complicated by the fact that China Tobacco—the largest manufacturer of cigarettes in the world—is owned by the Chinese government.^11^

Environmental characteristics are critical to consider when exploring factors that contribute to smoking. This is because health behaviour can be influenced by the environment in which people live.^12^ Indeed, recent studies that have examined the association between environmental determinants such as regional tobacco production and individual smoking behaviour^13–15^ have found positive associations. Yang *et al*, for example, reported that individuals residing in provinces with the highest rates of cigarette production in China were more likely to be current smokers as compared with individuals living in provinces with the lowest rates of cigarette production.^14^ Likewise, a meta-analysis conducted by Finan *et al*, showed a higher odds of adolescent smoking in the past month with increased tobacco outlet density around homes.^15^ Two systematic reviews published in 2020 and 2021 similarly revealed a positive association between tobacco outlet density and smoking behaviour.^16 17^

Given these findings, it is reasonable to hypothesise a positive relationship between regional tobacco production and SHS exposure, as well as retail tobacco outlet density and SHS exposure. Such evidence could help frame SHS as both an urgent public health problem and environmental hazard, which could open up new possibilities for intervention. Further, results could facilitate the identification of tobacco manufacturers and marketers who are directly responsible for these environmental problems, thereby increasing accountability and the level of awareness among the public. Unfortunately, to our knowledge, there are no studies that have explored these relationships. In light of this gap, the primary aim of this study is to examine the association between regional tobacco production and SHS exposure, as well as tobacco retail outlet density and SHS exposure.

A second aim is to explore possible mechanisms that explain the associations between regional cigarette production and SHS exposure as well as tobacco retail outlet density and SHS exposure. Our hypothesis is that the level of tobacco advertising will be higher in provinces and cities with more cigarette production and tobacco retail outlet density as these provinces are economically more reliant on the industry and might not adopt and/or strictly implement comprehensive tobacco control policies. The Hongta Group (a tobacco company in Yunnan province), for example, lobbied the provincial government to introduce policies that were advantageous for tobacco companies.^18^ Further, higher density of tobacco outlets will likely result in higher visibility of tobacco products, particularly since China’s law, in practice, does not apply to point of sale product display.^19^ Yang *et al*, for example, found that Changsha and Shenyang, two Chinese cities with large tobacco industry presence, had the highest levels of reported noticing tobacco advertising and sponsorship as compared with other Chinese cities (Beijing, Shanghai, Guangzhou and Yinchuan).^20^ Tobacco advertising can lead to increased tobacco consumption and uptake through influencing social norms; in other words, pervasive advertising can foster an environment where tobacco use is considered socially acceptable and less hazardous^21^ thus leading to more smoking and, consequently, leading more SHS exposure. According to the conceptualisation of Mead *et al* ‘social exposure to a behavior (defined as the composite of ways through which people see that behavior in their social, physical, and symbolic environments) can serve as a source of normative influence’ (p139).^22^

Findings can inform the consideration of the adoption and implementation of a national smoke-free law and other evidence-based policies to regulate tobacco manufacturing and marketing behaviour.

## Methods

### Study area and participants

This study employed a cross-sectional multistage sampling design. In stage 1, 21 cities, with over 1.5 million in population, were purposefully selected from across China and differentiated by regional location. Nine were located in eastern China, five in central China and the remaining seven in the west. About 68% of Chinese provinces were covered by the 21 cities in this study. In stage 2, two residential districts were randomly selected from the main urban zone of each city; new building districts and subdistricts were excluded. Subsequently, in stage 3, four communities were randomly selected within each residential district and in stage 4, 22 906 households were randomly selected in each community using a family household registration list. Individuals aged 15 years and older and who had lived in their home for at least a year were identified within each household. Finally, one eligible participant, determined by birth date closest to the contact date, was randomly selected from each household.^14^

### Data collection

Once an eligible individual agreed to participate in the study, a face-to-face interview was scheduled. During these interviews, participants were asked to fill out a structured self-administered questionnaire that took approximately 30 min. The same research protocol was used across all 21 cities to ensure standardisation of data collection.

### Measures

#### Dependent variable

The dependent variable in this study was SHS exposure and it was assessed through self-report. We defined SHS exposure as a non-smoker who reported daily exposure to SHS for at least 15 min/day at the time of the survey.^14 23^ SHS exposure was coded dichotomously as 2=exposure and 1=no exposure.

#### Individual-level independent variables

Sociodemographic characteristics such as age, gender, ethnicity, educational level, occupation and personal income were included. Personal income was reported as the average income per person in the household in the prior year.

Individual-level independent variables also included ‘exposure to tobacco advertising’. Respondents were asked whether they had seen any tobacco advertisements in the last 6 months. Responses were ‘never’, ‘seldom’, ‘sometimes’, ‘often’ and ‘almost always’ and coded as 1 through 5, respectively. Attitude towards SHS exposure was measured by the question, ‘“do you care that others smoke around you?’. Responses were ‘very’, ‘some’ and ‘do not care’ and coded as 1 through 3, respectively. Behaviour to prevent SHS exposure was measured by the question, ‘do you have any smoking restrictions in your household?’. Responses included ‘completely’, ‘partly’ and ‘no restrictions’, and were coded as 1 through 3, respectively.

#### Provincial and city-level independent variables

Four independent variables were included. Given that urban differences in smoking and SHS exposure are likely to reflect socioeconomic status^14^ controls were made for the level of economic development in the home province (per capita gross domestic product (GDP) in yuan). These data were obtained from the national population database.^24^ Given that women are less likely to be exposed to SHS, it is also important to know whether gender imbalance was related to tobacco production and retail intensity. As such, the city sex ratio was included, as measured by the proportion of men in the total population. Data were obtained from city governments’ websites for the year 2011 and were categorised as less than 50%, 50%– and more than 51.5%. Two measures related to the level of cigarette production at the provincial level were also included as the strength of the provincial tobacco industry is likely to be a crucial factor in influencing SHS exposure. These two separate, but closely related, measures were compared with test the reliability of the results. The first variable was estimated using cigarettes per ¥100 GDP (pieces/¥100 GDP) and was categorised as less than 5, 5–9 and 10 and more. The second variable was estimated using cigarettes per population (100 million/10 000 persons) and categorised as <500, 500–999 and 1000 and more. Data for both variables are collected by the national Tobacco Monopoly Bureau and available at the provincial level.^25^ The variable pertaining to tobacco retail outlet density in the cities was measured using number of tobacco retail outlets per 100 000 persons.^25^ This variable was categorised as less than 35, 35–39 and 40 and more^14^ (table 1).

**Table 1:** Demographic characteristics, regional variables and SHS exposure of study participants

Group	N	Sample (%)	Prevalence of SHS exposure	OR (95% CI)
**Age (years)**				
<25	2110	15.1	30.6	1.00
25–34	3061	20.8	27.7	0.87 (0.70 to 1.08)
35–44	2537	19.3	30.0	0.97 (0.77 to 1.22)
45–54	1620	21.0	29.0	0.93 (0.68 to 1.28)
55+	1878	25.5	24.8	0.75 (0.43 to 1.29)
**Gender**				
Male	3747	28.6	50.0	1,00
Female	7459	71.4	19.2	0.24 (0.08 to 0.69)**
**Ethnicity**				
Han	9776	89.7	30.0	1.00
Other	1430	10.3	11.0	0.29 (0.09 to 0.95)*
**Education**				
Elementary school or less	634	2.4	44.7	1.00
Junior high school	2390	18.3	50.9	1.28 (0.66 to 2.47)
High school	4089	37.4	27.2	0.46 (0.21 to 1.02)
Junior college or more	4093	39.0	16.0	0.24 (0.09 to 0.63)**
**Occupation**				
Managers and clerks	567	4.2	46.0	1.00
Professionals	580	4.1	42.7	0.88 (0.51 to 1.51)
Commerce and service	1254	5.3	53.5	1.35 (0.76 to 2.40)
Operations	2811	22.5	45.9	0.97 (0.47 to 2.14)
Students	1210	24.7	14.6	0.20 (0.07 to 0.55)**
Retired	1841	8.5	15.6	0.22 (0.08 to 0.64)**
Other	4144	25.	14.4	0.20 (0.06 to 0.65)**
Income/person/year (¥*)				
<10 000	1416	12.9	43.2	1.00
10 000–19 999	3130	29.8	42.9	0.99 (0.53 to 1.86)
20 000–29 999	3103	26.1	29.1	0.54 (0.28 to 1.03)
30 000–39 999	1413	14,6	10.5	0.16 (0.05 to 0.49)**
40 000–49 999	990	10.2	9.5	0.14 (0.04 to 0.45)**
50 000+	1154	11.5	15.4	0.24 (0.08 to 0.74)**
**Exposure to tobacco advertising**				
No	6561	54.0	18.1	1.00
Yes	4645	46.0	44.5	3.89 (1.75 to 8.68)**
**Regional variables**				
**Male % population**				
<50%	2991	36.7	38.7	1.00
50%–	2296	13.5	44.3	1.26 (0.64 to 2.45)
51.5%+	5919	50.8	16.2	0.31 (0.10 to 0.96)*
**GDP**				
<2000	3205	10.2	46.7	1.00
2000–2999	3560	42.6	33.3	0.57 (0.23 to 1.40)
3000+	4440	47.2	19.2	0.27 (0.09 to 0.80)*
**Cigarette production per unit population (100 million/10 000 persons)**				
<500	3909	26.7	27.8	1.00
500–999	4548	58.8	24.4	0.85 (0.74 to 1.03)
1000+	2749	14.5	43.0	2.06 (1.80 to 2.35)**
**Cigarette production per unit GDP (pieces/¥100 GDP)**				
<5	4437	35.9	26.7	1.00
5–9	4205	52.9	23.6	0.79 (0.63 to 1.03)
10+	2564	11.2	43.6	1.83 (1.31 to 2.55)**
**Tobacco retailer density (retailers/10 000persons)**				
<35	3273	46.7	26.2	1.00
35–39	2049	26.4	23.5	0.87 (0.41 to 1.88)
40+	5884	27.0	36.0	1.60 (1.37 to 2.12)**

*p<0.05; **p<0.01.

†Chinese yuan=US$6.5.

GDP, gross domestic product; SHS, secondhand smoke.

The above categorisations were implemented based on the principle and method of categorising continuous variables (p162)^23^ and prior practice.^14 26^ Moreover, we conducted the analysis using different categorisations of these variables and the results were very similar.

### Data analysis

Data were entered into a database using Microsoft Excel then imported into SAS (V.9.4) for statistical analyses. Descriptive statistics were used to calculate SHS exposure prevalence. Logistic regression models were carried out to examine the association between individual and regional-level variables and SHS exposure. Associations were confirmed through the application of a multilevel logistic regression model using the SAS NLMIXED procedure.^27 28^ Several models were built. The first was the ‘null’ model, a two-level (individual and city) model with random intercepts. No predictors were included except a constant to account for variation in SHS exposure across the 21 study cities. Using this base, we entered all individual, and provincial level GDP, as the basis model (model 1). Models 2, 3, 4 and 5 explored demographic characteristics, per capita cigarette production, per GDP cigarette production, as well as tobacco retail outlet density and their associations with SHS exposure (see table 2). All analyses were weighted^28^ with the use of sampling weights, non-participation weight and post-stratification weights (for more details see Yang *et al* 14).

**Table 2:** Multilevel logistic regression analysis (adjusted ORs and 95% CIs) of secondhand smoke exposure

	Null model	Model 1 (OR 95% CI)	Model 2 (OR (95% CI))	Model 3 (OR (95% CI))	Model 4 (OR (95% CI))	Model 5 (OR (95% CI))
**Gender**						
Male		1.00	1.00	1.00	1.00	1.00
Female		0.67 (0.42 to 0.77)**	0.54 (0.41 to 0.71)**	0.57 (0.41 to 0.78)**	0.54 (0.47 to 0.71)*	0.55 (0.43 to 0.70)**
**Occupation**						
Managers and clerks		1.00	1.00	1.00	1.00	1.00
Professionals		0.78 (0.67 to 0.92)*	1.01 (0.69 to 1.48)	0.88 (0.62 to 1.25)	1.12 (0.81 to 1.53)	1.17 (0.82 to 1.67)
Commerce and service		0.94 (0.79 to 1.12)	1.27 (0.98 to 1.65)	1.24 (0.96 to 1.60)	1.28 (0.80 to 2.06)	1.30 (0.76 to 2.24)*
Operations		0.73 (0.45 to 1.20)	0.90 (0.70 to 1.16)	0.89 (0.61 to 1.29)	0.96 (0.66 to 1.40)	0.90 (0.71 to 1.13)
Students		0.18 (0.06 to 0.57)**	0.26 (0.07 to 0.89)*	0.25 (0.07 to 0.82)^*^	0.29 (0.12 to 0.68)**	0.28 (0.11 to 0.74)*
Retired		0.34 (0.20 to 0.58)**	0.41 (0.19 to 0.87)*	0.41 (0.22 to 0.75)**	0.42 (0.20 to 0.89)*	0.41 (0.17 to.95)*
Other		0.27 (0.16 to 0.58)**	0.34 (0.14 to 0.79)*	0.35 (0.17 to 0.72)**	0.33 (0.14 to 0.75)**	0.31 (0.12 to 0.83)*
Income/person/year (in ¥*)						
<10 000		1.00	1.00	1.00	1.00	1.00
10 000–19 999		0.82 (0.36 to 1.87)	0.84 (0.45 to 1.60)	0.91 (0.47 to 1.79)	0.94 (0.55 to 1.60)	0.87 (0.51 to 1.48)^*^
20 000–29 999		0.67 (0.29 to 1.54)	0.65 (0.26 to 1.58)	0.71 (0.34 to 1.47)	0.75 (0.46 to 1.21)	0.63 (0.31 to 1.31)
30 000–39 999		0.25 (0.19 to 0.34)^**^	0.27 (0.18 to 0.41)**	0.28 (0.22 to 0.36)**	0.30 (0.22 to 0.47)**	0.27 (0.18 to 0.41)**
40 000–49 999		0.30 (0.21 to 0.42)**	0.33 (0.22 to 0.50)**	0.33 (0.23 to 0.47)**	0.36 (0.24 to 0.55)^**^	0.34 (0.35 to 0.54)**
50 000+		0.42 (0.37 to 0.49)**	0.45 (0.43 to 0.49)**	0.45 (0.40 to 0.51)**	0.45 (0.34 to 0.61)**	0.43 (0.35 to 0.54)**
**Exposure to tobacco advertising**						
No		1.00	1.00	1.00	1.00	1.00
Yes		2.56 (1.15.35.71)**	2.58 (1.10 to 6.07)*	2.75 (1.14 to 6.61)*	2.34 (1.05 to 5.28)*	2.39 (1.06 to 5.40)*
**Regional variables**						
***GDP***						
<2000		1.00	1.00	1.00	1.00	1.00
2000–2999		0.61 (0.57 to 0.65)**	1.08 (0.50 to 2.34)	0.95 (0.51 to 1.77)	0.50 (0.35 to 0.71)**	0.82 (0.36 to 1 to 85)
3000+		0.35 (0.20 to 0.64)**	0.55 (0.40 to 0.76)**	0.57 (0.43 to 0.76)**	0.25 (0.20 to 0.31)**	0.38 (0.23 to 0.62)**
**Cigarette production per unit population (100 million/10 000 persons)**						
<500			1.00			1.00
500–999			1.04 (0.71 to 1.52)			0.85 (0.47 to 1.54)
1000+			2.72 (1.56 to 4.76)**			2.01 (1.30 to 3.12)**
**Cigarette production per unit GPD (pieces/¥100 GDP)**						
<5				1.00		
5–9				0.81 (0.42 to 1.56)		
10+				1.69 (1.42 to 2.01)**		
**Tobacco retailers density (retailers/10 000persons)**						
<35					1.00	1.00
35–39					1.01 (0.30 to 3.40)	0.87 (0.23 to 3.31)
40+					2.66 (1.62 to 4.38)**	2.32 (1.39 to 3.86)*
Random parameters between regions	3.12**	3,09**	2.15*	2.03*	1.81*	1.64*
Fixed parameters	2.92**	2.29*	2.26*	2.24*	2.14*	2.09*

*p<0.05; **p<0.01.

†Chinese yuan=US$6.5.

GDP, gross domestic product.

Subsequently, structural equation modelling was employed to determine possible mechanisms that explain the associations between regional cigarette production and SHS exposure as well as between tobacco retail outlet density and SHS exposure. The CALIS procedure in SAS was used for the structural equation analysis. The dependent variable was SHS exposure, indirective variables were tobacco advertising, attitude and behaviour to prevent SHS exposure, and independent variables were regional tobacco production and tobacco retail outlet density. The maximum likelihood method estimates the parameter values of a statistical model; χ^2^ (p<0.05), Goodness-of-fit index(GIF) (>0.9) and Standized Root mean square residual (<0.1) were used to assess the quality of the model.^29^

## Results

### Demographic characteristics

A total of 18 875 individuals were identified as potential subjects for this study, of which 17 424 (92.3%) were successfully contacted and agreed to participate in the survey. Of the 17 424 surveys obtained, 16 866 (96.8%) were complete and valid and of these 11 206 were from non-smokers and, thus, included in this study.

Results showed that 15.1% of the participants were less than 25 years of age, 20.8% were between 25 and 34, 19.3% were between 35 and 44, 21% were between 45 and 54 and 25.5% were 55 years or older. The majority were also women (71.4%), Han (89.7%) and had at least a high school degree (76.4%). Almost a quarter of the participants worked in operations (22.5%) or were a student (24.7%). A little less than 30% of the participants had an income of between ¥10 000 and ¥19 999 a year and 26.1% had an income of between ¥20 000 and ¥29 999 a year (table 1).

### Cigarette production and retail density

Just over half (58.8%) of participants were living in medium (500–999 million/10 000 persons) cigarette production provinces and 14.5% were living in high level (1000+ million/10 000 persons) cigarette production provinces. When measuring cigarette production per unit GDP, 52.9% and 11.2% of the participants were located in medium (5–9 pieces/¥100 GDP) and high level (10+ pieces/¥100 GDP) cigarette production provinces, respectively. Moreover, 26.4% and 27.0% were residing in medium (35–39 retailers/10 000 persons) and high level (40+ retailers/10 000 persons) tobacco retailer density cities, respectively (table 1).

### Prevalence of exposure to SHS

The overall prevalence of SHS exposure was 28.1% (95% CI 27.1 to 29.0). Table 1 shows that exposure to SHS was significantly associated with gender, ethnicity, education, occupation, income, exposure to tobacco advertising, regional GDP, province-level per capita cigarette production and per GDP cigarette production, and city-level gender imbalance and tobacco retail outlet density.

### Factors associated with SHS exposure

Table 2 shows the results of the multiple logistic regressions(Model 1). SHS was significantly associated with gender, occupation and income. Women (OR: 0.67) were less likely to be exposed to SHS than men. Likewise, students (OR: 0.18) and retired people (OR: 0.34) had lower odds of SHS exposure versus managers and clerks. Compared with low-income subjects, those with middle and higher income (¥30 000–¥39 999; ¥40 000–¥49 999 and ¥50 000 or more) had higher odds of SHS exposure (OR: 0.25, 0.30 and 0.42, respectively).

The variables measuring the level of cigarette production at the provincial level, and tobacco retail density were added to models 2–5. Results showed that the province-level cigarette production per capita (OR: 2.72 (95% CI 1.56 to 4.76)), and cigarette production per GDP (OR: 1.69 (95% CI 1.42 to 2.01)) were significantly associated with SHS exposure. The odds of SHS exposure were also higher in cities with a greater density of tobacco retail outlets (OR: 2.66 (95% CI 1.63 to 4.38)) (table 2).

Significant positive correlations were found between regional-level per capita cigarette production (r: 0.54810, p=0.0001), per GDP cigarette production (r: 0.49077, p<0.0001), city-level tobacco retail outlet density (r: 0.06920, p<0.0001) and city-level SHS exposure prevalence.

**Figure 1:**
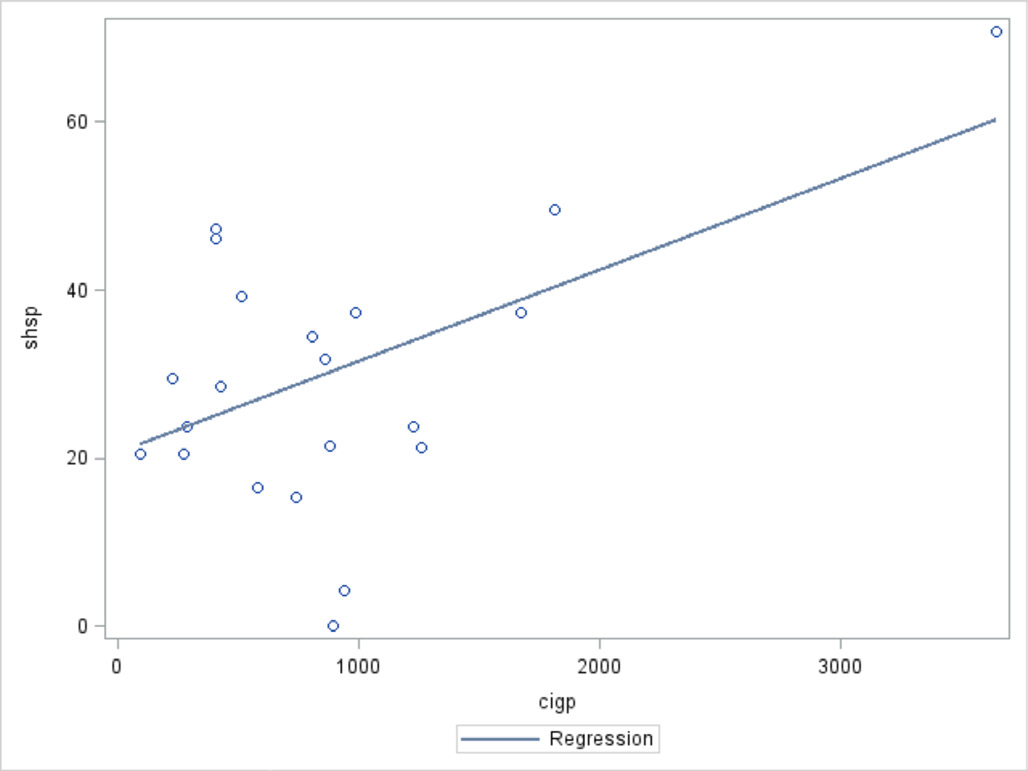
Regional-level cigarette production per unit gross domestic product and regional-level prevalence of secondhand smoke (SHS) exposure.* *cigp, regional-level cigarette production per unit population; shsp, regional-level SHS exposure prevalence. r: 0.54810; p<0.0001.

**Figure 2:**
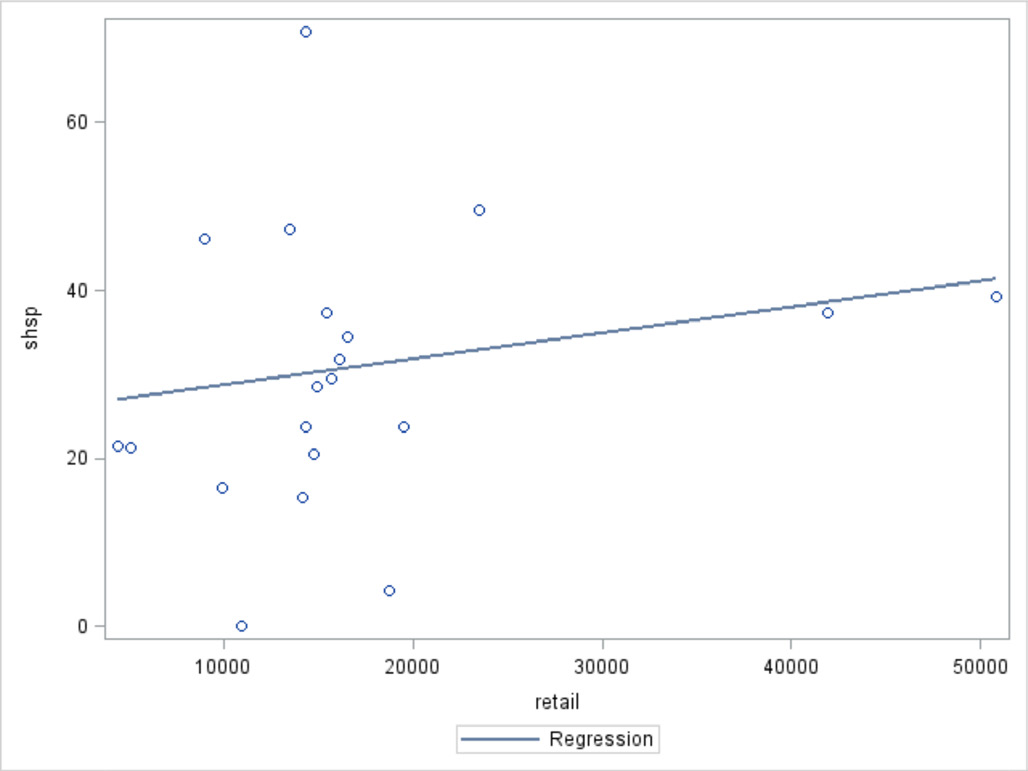
Regional-level cigarette production per unit gross domestic product (GDP) and regional-level prevalence of secondhand smoke (SHS).* *cigg, regional-level cigarette production per unit GDP; shsp, regional-level SHS exposure prevalence. r: 0.49077, p<0.0001.

**Figure 3:**
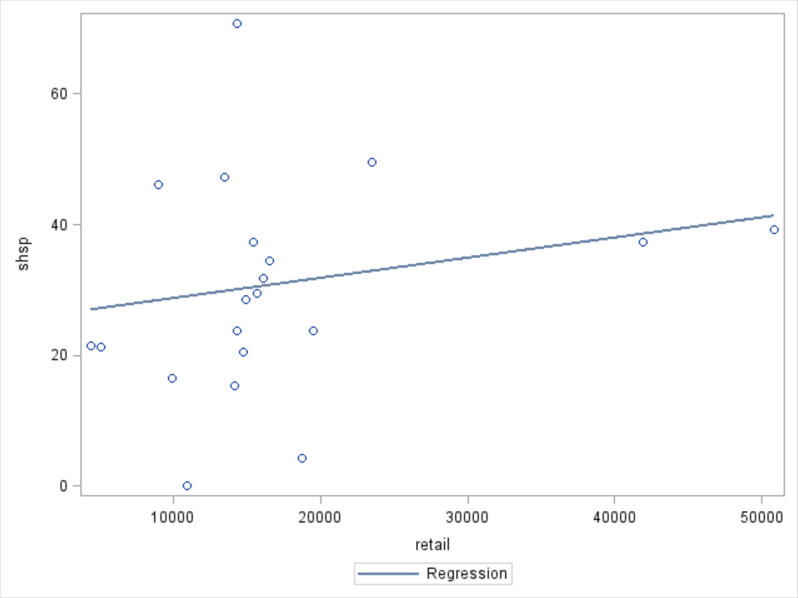
City-level tobacco retailer density and city-level secondhand smoke (SHS) exposure prevalence.* *retail, city-level tobacco retailer density; shsp, city-level SHS exposure prevalence. r: 0.06920, p<0.0001.

Figures 1–3 show that the prevalence of provincial level SHS exposure increases as regional-level per capital cigarette production and per GDP cigarette production, and city-level tobacco retail outlet density increases.

**Figure 4:**
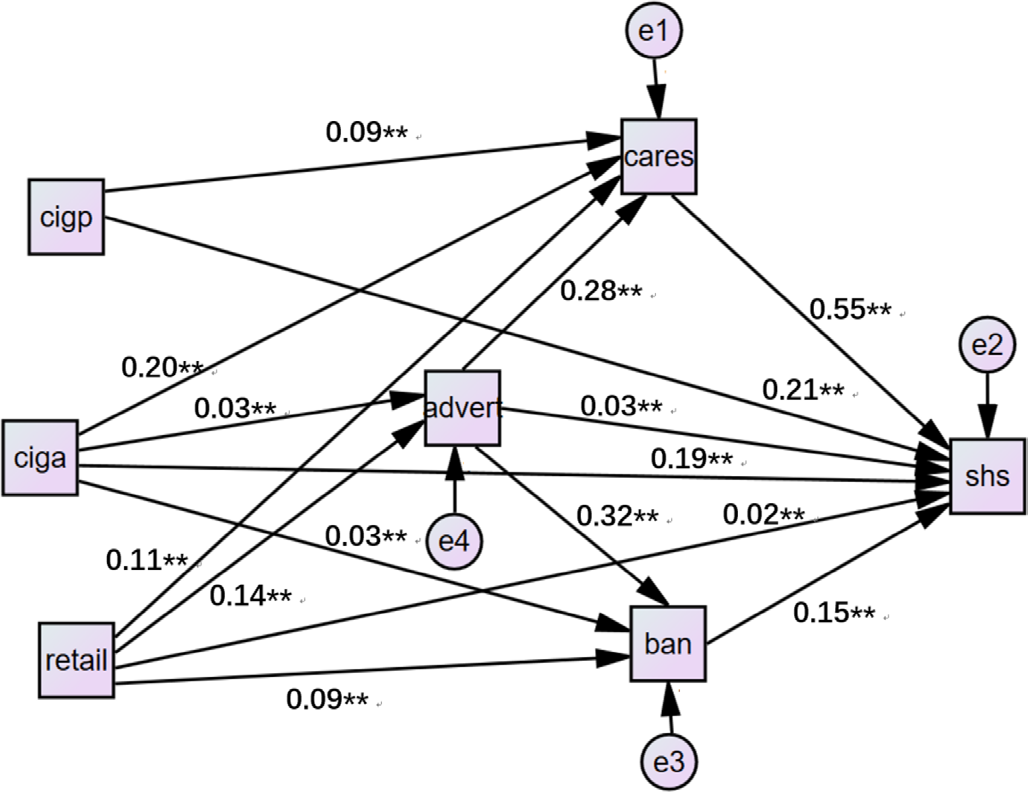
Structural equation analysis. Adver, exposure to tobacco advertising; Ban, behaviour to prevent; Cares, attitude towards secondhand smoke (SHS) exposure; Ciga, cigarette production per unit gross domestic product (GDP); Cigp, cigarette production per unit population; Retail, tobacco retailer outlet density; SHS; SHS exposure.

Table 3 shows the mean and SD of variables included in the structural equation model. Structural equation analysis showed goodness of fit (χ^2^: 1.13, p=0.323; Goodness-of-fit inde(GIF) : 0.986, Standized Root mean square residual(REM): 0.004). Each regional tobacco-related variable was found to have direct influence on individual SHS exposure (figure 4): the β value was 0.19 (p<0.01) in the province-level cigarette production per unit GDP path; the β value was 0.21 (p<0.01) in the cigarette production per unit population and the β value was 0.02 (p<0.05) in city-level tobacco retailer density path. The province-level cigarette production per unit population had an indirect influence on SHS exposure through individual attitude to prevent from SHS (β=0.09 (p<0.01) and β=0.55 (p<0.01)). The province-level cigarette production per unit GDPand city-level tobacco retailer density can influence SHS exposure though exposure to tobacco advertising and attitude and behaviour to prevent SHS exposure. The two β values were 0.03 (p<0.01) from cigarette production per unit GDP and tobacco retailers density to exposure to tobacco advertising. β values were 0.28 (p<0.01) and 0.32 (p<0.01) from exposure to tobacco advertising to attitude and behaviour to prevent SHS exposure. β values were 0.55 (p<0.01) and 0.15 (p<0.01) from attitude and behaviour to prevent SHS exposure to SHS exposure.

**Table 3:** Means and SD of variables included in construction model

Variables	Means	95% CI	Value range
Cigarette production per unit GDP (Ciga)	47 238.33	46 895.04 to 47 581.62	24 231–1 12 372
Cigarette production per unit population (Cigp)	808.45	797.09 to 819.81	95–3649
Retail	19 739.89	19478.55 to 20001.19	1464–50835
Exposure to tobacco advertising (Adver)	2.67	2.64 to 2.68	1.00–5.00
Attitude towards SHS exposure (Cares)	2.32	2.31 to 2.34	1.00–4.00
Behaviour to prevent SHS exposure (Ban)	2.42	2.40 to 243	1.00–3.00
SHS exposure (SHS)	1.28	1.27 to 1.29	1.00–2.00

Adver, exposure to tobacco advertising; Ban, behaviour to prevent; Cares, attitude towards SHS exposure; Ciga, cigarette production per unit gross domestic product; Cigp, cigarette production per unit population; Retail, tobacco retailer outlet density; SHS, secondhand smoke.

## Discussion

This study contributes to the literature in four important ways. First, we found that nearly one-third (28.1%; 95% CI 27.1 to 29.0) of the study participants were exposed to SHS despite the use of a stricter definition for SHS exposure (ie, daily exposure to SHS for at least 15 min/day) than other studies in China.^30 31^ This indicates that currently SHS is still a serious public health problem in China and a great challenge to the public’s health. Interestingly, findings revealed that women were significantly less likely to be exposed to SHS than men. This could be related to norms against female smoking^10 32^ in Chinese society and that men tend to socialise with other men, many of whom are smokers, thereby increasing exposure to SHS.

Second, to our knowledge, this is the first study to explore the relationship between regional tobacco production and SHS exposure. Findings showed that the prevalence of SHS exposure is higher in regions with more cigarette production. There also appears to be a dose response; the prevalence of SHS exposure was statistically significantly higher in regions with the highest production as compared with regions with the lowest. Several existing studies have already found a significant association between regional tobacco production and smoking.^14^

Third, this study also contributes to literature by exploring the relationship between tobacco retail outlet density and SHS exposure. Findings showed that the prevalence of SHS exposure was found to be 26.2%, 23.5% and 36.0% in low, middle and high tobacco retail outlet density groups, respectively; there was a statistically significant difference between the highest density group and reference group (lowest density group) (OR: 2.66 (95% CI 1.62 to 4.38)). These results are supported by existing research, which has indicated that tobacco retail outlet density is associated with smoking.^17^

Finally, this study also explored possible mechanisms that explain the associations between regional cigarette production and SHS exposure as well as tobacco retail outlet density and SHS exposure. Results showed that these associations may be explained by the level of tobacco advertisement, which then influenced attitude and behaviour towards smoking and SHS exposure. Currently, in China, the tobacco advertising, promotion and sponsorship law is not comprehensive. While advertising is banned in mass media, public places, public transport and outdoors, other advertising activities such as sponsored events, promotional discounts and retailer incentive programmes are allowed.^19^ There is also evidence to show that existing bans are not well implemented. Yang *et al*, for example, found that 37.2% of respondents in six Chinese cities reported yes to noticing things that encourage smoking in the previous 6 months.^20^

SHS exposure contributes to many diseases, including lung cancer, heart disease and asthma in children.^2 4^ Accordingly, it constitutes a very serious environmental problem, especially in indoors areas. This study clearly underscores the responsibilities of tobacco manufacturers and retailers for these environmental problems and the importance of holding these entities accountable along with the national and provincial governments. Despite China’s system of centralised control over the development of the tobacco industry, provincial governments also play a key role and are considered important stakeholders. These provincial governments have tended to focus on regional economic development at the expense of addressing the issue of smoking and instituting strong tobacco control measures.^14^

### Implications

This study provides new knowledge about the environmental factors associated with SHS exposure in urban China. SHS exposure is not just an individual health awareness and behavioural problem but also the responsibility of cigarette manufacturers and retailers. To curb the escalating health and economic cost of tobacco use in China, it will be critical for the country to enact and fully enforce a national smoke-free law as per the FCTC. The government—both national and provincial—should prioritise people’s health and change the current economic reliance on the tobacco industry. Restricting the permitted density of tobacco retail outlets, cigarette production and marketing of tobacco products in China will also be critical. Policy solutions for restricting the density of tobacco retail outlets include retail licensing laws, imposing a limit on the number of retailers in a geographic area, and banning sales in specific types of retailers and locations where youth frequent.^33^ While China’s Tobacco Monopoly Law mandates retail licensing and prohibits the issuance of licenses to vendors around primary and secondary schools, it does not specify the distance within which tobacco sales are banned.^19^ Moreover, the law also does not ban or restrict sale of tobacco products around playgrounds, stadiums/arenas, in healthcare facilities or in cultural facilities.^19^ These policy approaches will be important for China to consider to address the current situation.

While this study was conducted in China, the finding that environmental factors (province-level per capital cigarette production and city-level tobacco retail outlet density) were associated with SHS exposure could potentially be extendable to other similar countries, thereby underscoring the importance for these countries to also consider policy approaches that address environmental determinants. More research, however, is also needed in these other international contexts to further explore the relationship between regional tobacco production and SHS exposure, and retail tobacco outlet density and SHS exposure.

### Study strengths and limitations

The strengths of this paper are mainly its large sample size and its high level of representativeness (the survey covered 21 cities across China mainland). There are, however, some limitations to this study. First, we used a cross-sectional study design, which limits causal inference; the factors identified—namely cigarettes production and density of tobacco retail outlet—can only be considered contributing factors to SHS exposure. However, we did employ a large sample, and our findings met several criteria for inferring causality, including the strength of some associations, multiple consistent results, theoretical support for the plausibility of effect and uncovering the mechanism within the associations. Longitudinal studies are needed to confirm the directionality and causality of the reported associations. Second, only urban residents were included in this survey. Thus, our results are not generalisable to the overall population of China, which has a very substantial rural component. Third, it might be possible that other factors such as variations in subnational tobacco control policies, including smoke-free policies, and the enforcement of such policies, might influence with what the outcome was. Future studies could explore this by examining data related to these policies, including the level of enforcement of these policies. Finally, cigarettes produced in a province is allowed to be sold in other provinces in China. While, we were not able to adjust for this factor, the majority of smokers in China smoke their locally produced brands. This is because each province produces cigarettes that suit the taste and culture of local consumers.^34 35^ Furthermore, there are strict restrictions on the inter-regional transportation of tobacco according to the Tobacco Monopoly Law of the People’s Republic of China.

**What this paper adds?**

- Environmental characteristics are critical to consider when exploring factors that contribute to smoking. This is because health behaviour can be influenced by the environment in which people live.
- Recent studies that have examined the association between environmental determinants such as regional tobacco production and individual smoking behaviour have found positive associations. However, to our knowledge, no studies have explored the relationship between regional tobacco production and secondhand smoke (SHS) exposure.
- This study found that two key environmental factors (province-level per capital cigarette production and city-level tobacco retail outlet density) were associated with SHS exposure in China. Results also showed that these associations may be explained by the level of tobacco advertisement, which influences social norms, including attitudes and behaviours toward SHS exposure.

## Data Availability

Data are available upon reasonable request.
